# The fat mass and obesity-associated (FTO) gene allele rs9939609 and glucose tolerance, hepatic and total insulin sensitivity, in adults with obesity

**DOI:** 10.1371/journal.pone.0248247

**Published:** 2021-03-08

**Authors:** Ann Kristin Hjelle de Soysa, Mette Langaas, Anida Jakic, Fariba Shojaee-Moradie, A. Margot Umpleby, Valdemar Grill, Ingrid Løvold Mostad

**Affiliations:** 1 Department of Clinical Nutrition and Speech-Language Therapy, Clinic of Clinical Services, St. Olavs Hospital - Trondheim University Hospital, Trondheim, Norway; 2 Faculty of Medicine and Health Sciences, Department of Clinical and Molecular Medicine, Norwegian University of Science and Technology, Trondheim, Norway; 3 Faculty of Information Technology and Electrical Engineering, Department of Mathematical Sciences, Norwegian University of Science and Technology, Trondheim, Norway; 4 Faculty of Medicine and Health Sciences, Norwegian University of Science and Technology, Trondheim, Norway; 5 Faculty of Health and Medical Sciences, University of Surrey, Guildford, United Kingdom; Medical University of Vienna, AUSTRIA

## Abstract

The objective of the study was to assess associations of the rs9939609 FTO allele to glucose tolerance, hepatic and total insulin sensitivity (IS) in individuals with obesity. From a low-dose hyperinsulinemic euglycemic clamp with glucose-tracer, hepatic IS was assessed by rates of basal and suppressed glucose appearance (Ra), a measure of endogenous glucose production (EGP), and the hepatic insulin resistance index (HIR). Total IS was assessed by rates of glucose infusion (GIR), disappearance (Rd), and metabolic clearance (MCR). From a meal test we assessed IS by the Matsuda index and glucose tolerance by glucose and insulin measurements in the fasted state and postprandially for 2.5 h. The meal test was performed in 97 healthy individuals with BMI ≥35 in similar-sized risk-allele groups (n = 32 T/T, 31 A/T, and 34 A/A), and 79 of them performed the clamp. We analyzed outcomes separately for males and females, and adjusted glucose Ra, Rd, MCR, GIR, and HIR for fat mass. We did not find genotype effects on EGP. Among males, genotype A/A was associated with a significantly lower glucose Rd, MCR, and Matsuda index score relative to genotype T/T. Glucose tolerance was significantly lower in males with genotype A/T vs. T/T and A/A. For females, there were no genotype effects on hepatic or total IS, or on glucose tolerance. Independently of genotypes, females displayed a significantly better hepatic and total IS, and better glucose tolerance than males. We conclude that in subjects with similar obesity we did not register any FTO risk-allele effect on hepatic IS. A FTO risk-allele effect on total IS was registered in males only, findings which need to be reproduced in further studies. Results confirm marked differences in IS between the biological sexes and extend present knowledge by demonstrating a lower endogenous glucose production in females vs. males in uniformly obese individuals.

## Introduction

The risk allele SNP rs9939609 of the fat mass and obesity-associated gene (FTO) is well known for its association with obesity and obesity associated traits such as insulin resistance and type 2 diabetes mellitus (T2DM) [1–3]. Animal studies suggest that FTO plays a role in beta cell function [4–6], and it may also play an important role in the regulation of gluconeogenesis [7]. Since abnormal glucose output (chiefly from the liver) and clearance are key features of T2DM, assessing the impact of FTO in humans on these parameters is of clinical interest.

Associations between the FTOs risk allele and glucose homeostasis have been tested using different methods in populations differing in age, sex, ethnicity, and body mass index (BMI) [8–21]. The use of different study populations and methods have made literature comparisons difficult, and studies in humans have yielded inconsistent results on FTOs effect on parameters of glucose homeostasis [9–22]. Non-genetic differences in phenotypes of risk- and non-risk carriers because of body size heterogeneity most likely play a role; and divergences might stem from the confounding influence of overweight *per se*. To minimize the risk of weight diverging influences our study was designed for healthy obese adults (BMI ≥ 35) only, and to increase the statistical power a double-blind selection of genotypes was performed to get the same number of heterozygotes and homozygotes for the risk and non-risk alleles of FTO.

We used the euglycemic clamp technique with concomitant measurements (by tracer technology) of endogenous glucose production (EGP) and uptake (glucose Rd). Further, we investigated insulin sensitivity (IS) and glucose tolerance as glucose and insulin responses to a test meal.

Our principle aim was to clarify in obese individuals whether the FTO risk allele is associated with reduced hepatic IS, while secondary aims were to test the FTO risk alleles association to glucose tolerance and total IS, taking into account the effects of biological sex.

## Materials and methods

### Participants and study design

**Fig 1:**
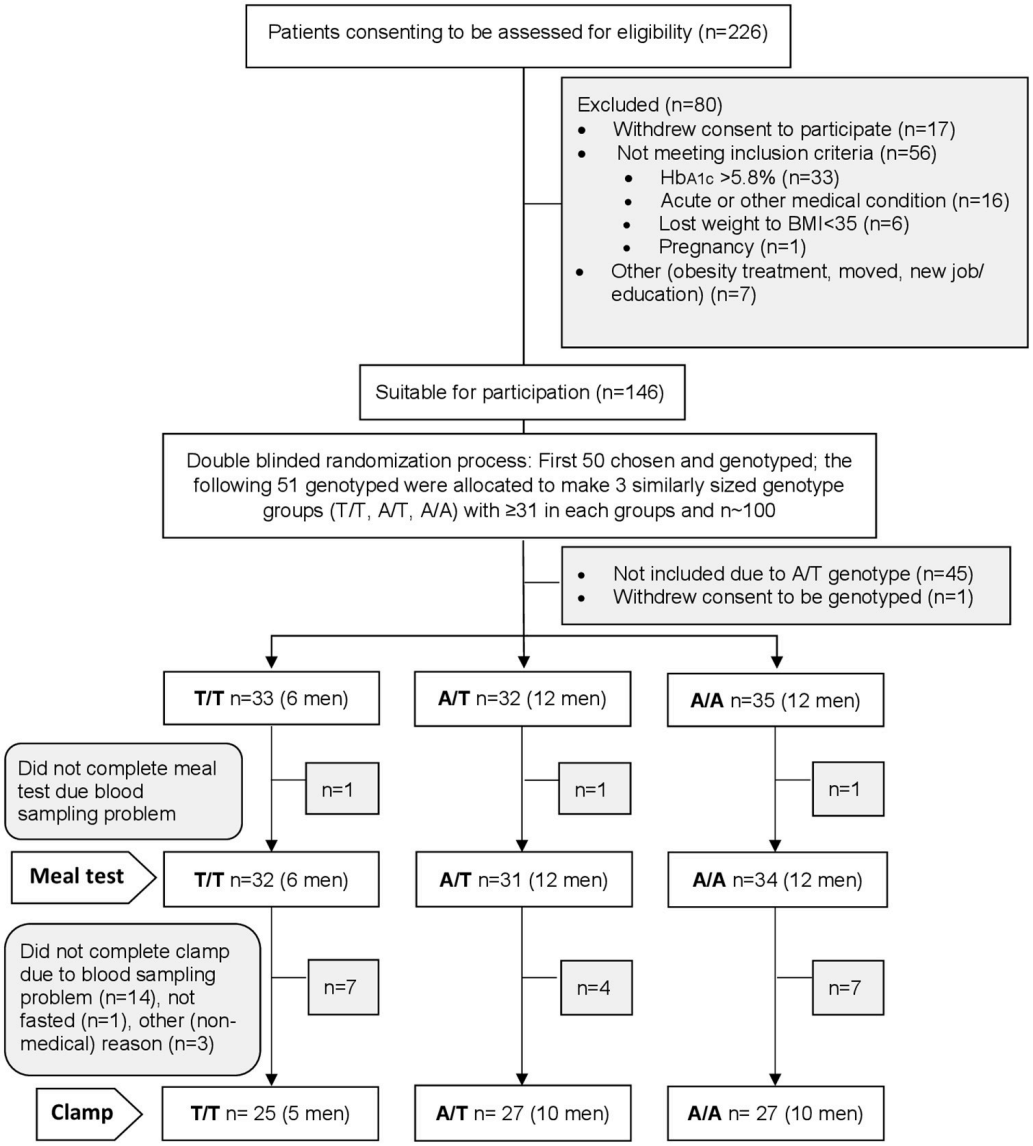
Flow diagram, enrollment and group allocation.

Between 2013 and 2015 adults 20 y or older with BMI ≥35 kg/m^2^ newly referred to the hospital’s out-patient obesity clinic, were invited to participate in a cross sectional metabolic and genetic study. Enrollment and group allocations are described in Fig 1. Exclusion criteria were a confirmed diabetes diagnosis or glycated hemoglobin (HbA1c) >5.8% (40 mmol·mol^-1^), pregnancy, cardiovascular incidents within the last 4 years, or taking anticoagulants.

Participant selection was done blinded to participants and investigators. The first 50 participants were included according to the inclusion criteria, only, with no focus on genotype. In the Norwegian population study, HUNT, the frequency of the FTO risk (minor) allele rs9939609 among overweight individuals was found to be 0.44 [23], and assuming Hardy-Weinberg equilibrium this gives genotypes probabilities for genotype T/T 0.31, A/T 0.49, and A/A 0.19. To guard against not obtaining adequate numbers of homozygotes for three similarly sized groups, an external controller selected the last 50 participants among the eligible volunteers so that mainly homozygotes were included.

Each participant was tested during two full days, one week apart. The median (25th, 75th percentiles) time from consent to the first test day was 5.0 (3.4, 6.6) months; and it took 2 y and 4 months to complete the study. The regional ethics committee approved the study (Ref. 2013/642). All volunteers gave their signed informed consent. The study was conducted according to the guidelines of the Declaration of Helsinki.

### Genotyping

DNA was extracted from peripheral blood leukocytes from EDTA whole blood using the Gentra Purgene Blood Kit (QIAGEN Science, Germantown, MD, USA). The genotyping of rs9939609 FTO was performed using 7900HT Fast Real-Time PCR System and predesigned TaqMan SNP Genotyping Assays (Life Technologies/ Thermo Fisher Scientific, Waltham, MA, USA) specified for the SNP. A positive and a negative control were included on each sample tray [24].

### Glucose tolerance

We tested glucose tolerance to a standardized real-life mimicking meal. Participants were instructed orally and in writing to be sedentary (allowed to walk at a leisurely pace) and avoid alcohol in the 24 h preceding the meal test. The participants arrived at 0815 h in the morning after 10 h of overnight fasting, including a temporary discontinuation of medications and tobacco-products. They consumed a standardized meal of 600 kcal (2502 kJ, 48% carbohydrates, 17% protein and 35% fat) within 15 min. The meal consisted of whole grain bread, butter, cheese, jam, orange juice, and either milk or sweetened yoghurt drink [25].

Blood was sampled in EDTA tubes in the fasting mode and then half-hourly for 150 min. Samples were centrifuged immediately (2110 RCF, 10 min, 18° C). Plasma, frozen in cryo tubes, were kept at -80° C pending analyses of glucose and insulin. Plasma glucose was measured using Biosen C-Line Clinic/GP+ (EKF Diagnostics, Cardiff, UK) and immunoreactive insulin using human insulin-specific RIA kits (HI-14K, Merck Millipore, Merck KGaA, Darmstadt, Germany).

### Insulin sensitivity (IS)

Hepatic IS was assessed by a 2 h hyperinsulinemic euglycemic low-insulin infusion (0.3 mU·kg^-1^·min^-1^) clamp with concomitant infusion of a [6, 6-^2^H_2_] glucose tracer. The participants arrived in the morning after an 11 h overnight fast abiding by the same instructions for fasting as to the meal test detailed above. They were instructed to remain sedentary for the last 48 h preceding the clamp. Additionally, we instructed participants to consume a specified low fat sandwich based meal at home no later than 2200 h the night before the test. To ensure that they ate the same they were provided a photo of the meal and written instructions regarding which foods and amounts they should eat. The meal provided 2502 kJ (600kcal) and had a calculated energy contribution of 57%, 15% and 28% from carbohydrate, protein and fat, respectively.

Upon arrival, a cannula was inserted into each antecubital fossa vein, one for blood sampling, the other for administration of infusates. Participants stayed semi-supine during the test.

Blood samples were taken for the determination of unenriched glucose (at -5 and 0 min). After a priming dose of 170 mg, a primed infusion of [6, 6-^2^H_2_] glucose (1.7 mg.min^-1^) was initiated at time 0.

After equilibration with [6, 6-^2^H_2_] glucose four samples were taken to measure baseline glucose metabolism from 100 to 120 min. Before the low-insulin infusion of insulin Actrapid started at 120 min the line was primed by running the pump at 70 ml/h for 20 sec after which the infusion rate was reduced to 7 ml/h for the 2 h clamp (min 120–240).

Blood samples were collected every 5 min during the clamp in order to swiftly measure the glucose concentration by the Biosen C-Line equipment. An infusion of dextrose (20%) spiked with [6, 6-^2^H_2_] glucose (8 mg·g^-1^ glucose) was adjusted between 120 min-240 min to achieve euglycemia, every 5 min as needed.

Blood samples were also taken for determination of glucose, insulin concentrations, and glucose enrichment. Sampling times were at min 0, 100, 110, 115, 120, 150, 180, 210, 220, 230, and 240. Samples for determination of glucagon were taken at min 100 and 240. Samples were centrifuged at 997 RCF, 10 min, 4° C, separated into three aliquots and frozen immediately in cryo tubes at -80° C, pending later analyses. The insulin infusion was stopped at 240 min whereas the glucose infusion continued for another 30 min.

Duplicate samples of insulin and pre-spiked and spiked glucose infusions were kept for analysis of concentration and enrichment, respectively. Plasma insulin and glucagon were analyzed by RIA (HI-14K and GL 32K Merck Millipore, Merck KGaA, Darmstadt, Germany).

Isotopic enrichment of plasma glucose [26] was determined by gas chromatography-mass spectrometry (Agilent Technologies 5975C inert Xl EI/CI MSD, Santa Clara, CA, USA). The plasma glucose concentration and enrichment time-courses were smoothed using optimal segments analysis [27].

### Calculations

Clamp success was tested by dividing mean basal and mean clamped glucose values, respectively, by 4.5 mmol·L^-1^ (the average of the target euglycemic glucose range of 4.0–5.0 mmol·L^-1^). We considered accuracy as acceptable if mean glucose values were within +/-10% of the mean of the target range of 4.0–5.0 mmol·L^-1^. Endogenous glucose production (Ra) and glucose uptake (Rd) (μmol·min^-1^·kg^-1^) were calculated using Steele’s non-steady-state equations [28], modified for stable isotopes, assuming a volume of distribution of 22% body weight. The calculation was also modified for inclusion of [6,6-^2^H_2_] glucose in the dextrose infusion [29]. The hepatic insulin resistance index (HIR) [30,31] was calculated as the product of basal glucose Ra (μmol·min^-1^·kg^-1^) and fasting, i.e. basal insulin concentration (average of minutes 100, 110, 115, and 120) (pmol·L^-1^). A high value of HIR means high insulin resistance. Glucose MCR was calculated from glucose Rd/glucose concentration. Exogenous glucose infusion rate (GIR), was calculated for each participant by averaging the values in the steady state in the end of the clamp (minutes 220, 230, and 240). Clamp variables were normalized by kg fat-free mass (FFM) [32].

From the meal test whole body insulin sensitivity was estimated by the Matsuda index [30] where the average of glucose and insulin were values from time points 0, 30, 60, 90, 120, and 150 min. A high value of the Matsuda index means high insulin sensitivity.

Based on power calculations performed in an earlier study [33] a potential 15% difference in IS was expected to be detected between the unpaired dominant genetic groups (80% power, *p*-value cut-off 0.05) with a sample size of at least 31 individuals in each of the FTO rs9939609 risk allele groups (T/T, A/T, and A/A). The aim was a sample totalling100 participants.

### Anthropometry and body composition

The participant’s height was recorded to the nearest 0.1cm and weight (in underwear) to the nearest 0.1kg (Seca 285 wireless measuring station, Hamburg Germany). Waist and hip circumferences were measured manually. Body composition was measured using two techniques: direct segmental multi-frequency bioelectrical impedance (DSM-BIA) (InBody720, Biospace Co., Ltd., Seoul, Korea) and by dual energy x-ray absorptiometry (DXA) (Holigic, Inc., Apex Software, Bedford, MA, USA). The participant’s DXA scan of the body included head, trunk and legs. We did not scan the arms in full because the size of the bench was too small. Therefore, we did not use upper extremities in the analyses. Compartmentalization of the body without the arms was performed in a standardized way to avoid in-between subject differences due to inaccurate measurements. The DXA scan also included waist, but not hip circumference. Due to difficulty in manual measurement of waist circumference in the participants, the DXA measurements are reported for the waist.

### Statistical analyses

The statistical analyses were performed using R language [34] and Stata [35]. To ensure that an effect of FTO is not uncovered because of biological sex differences we kept our tests exclusively within the two sex groups. We present summary statistics as medians, 25th and 75th percentile values due to the presence of outliers and non-normal responses. Accordingly, we used mainly robust and non-parametric statistical methods for the data analysis. For one-way analysis of variance (ANOVA) the Kruskal-Wallis method and reported *p*-values were used. We used the Wilcoxon-Mann-Whitney two-sample test for analyzing sex differences, and the chi-squared test for comparison of proportions between two groups. A *p*-value below 0.05 is considered significant.

Associations between the FTO risk allele genotype of the individuals and meal and clamp responses were tested with regression analyses.

Robust multiple linear regression (robust linear model, RLM) was performed for one-time responses (only genotype as covariate), based on the so-called M-estimation (a robust method for reliable statistical analysis under a “broad class of distributions” [36]), as implemented in the function ‘rlm’ in the MASS package in R [37]. Linear mixed effects models (LMM) were used for repeated measures responses for each individual before and after test meal, or insulin infusion during clamp (denoted time). In the model the effect of time is moderated by genotype (denoted genotype and genotype-time interaction). Correlation between each participant’s measurements is ensured by allowing each individual to have his/her own random intercept and fitted using the R package ‘lme4’ [38].

For all analyses genotype had codominant coding, that is, a factor with three levels denoted by the number of minor risk alleles present, thus 0, 1, or 2 for genotypes T/T, A/T, and A/A, respectively. Area under curve was calculated using the trapezoid rule.

Moreover, instead of statistical methods that make distributional assumptions we used bootstrapping [39] (nicely explained in [40]) which is a method that uses our original data to produce numerous random samples from which we construct 99% bootstrap confidence intervals (CI) for the regression parameters and selected contrasts (difference in regression coefficients). For the RLM the residual method was used (function ‘Boot’ in the R package car [41]) with 50000 bootstrap replications and the bias-corrected accelerated (BCa) interval method. For the LMM, restricted maximum likelihood estimation was applied in combination with parametric residual bootstrap with 50000 bootstrap replications and the percentile interval method. Statistical significance of a regression parameter or contrast was assumed if a 99% bootstrap CI did not include the hypothesized value (mostly 0). To address the multiple testing burden assessment of significance is based on 99%.

## Results

### Characteristics of the study participants

A total of 97 participants (67 women and 30 men), median (25th, 75th percentile) age 43 (32, 50) y, weight 120.9 (109.7, 142.3) kg and BMI 42.8 (39.5, 46.5) kg·m^-2^ participated in the current study with a meal test of whom 79 completed the clamp. Not completing the meal test or the clamp were mainly due to blood sampling problems, see Fig 1 for details. Anthropometric and body composition measures by genotype stratified by sex are shown in Table 1. There were no differences in BMI or weight between genotype groups; however, among men, there were significant differences between the genotype groups in age, android:gynoid fat ratio, and visceral fat mass. Females had lower android:gynoid fat ratio than men (median 0.53 vs. 0.86, respectively, p<0.0001) and visceral fat mass (median 757 g vs. 939 g, p = 0.008), independent of genotype. On an overall level, measurements in the meal and clamp tests differed between males and females. To account for sex differences in IS we performed sex-stratified analyses.

**Table 1:** Anthropometric and body composition measures by sex and genotype group, *n* = 97.

	T/T	A/T	A/A
	*n* = 6 male, 26 female	*n* = 12 male, 19 female	*n* = 12 male, 22 female
**Age (years)**			
males*	28.0 (25, 31)	41 (37, 54)	44.5 (37, 51)
*females*	43.5 (33, 48)	45 (37, 51)	42 (29, 53)
**BMI**^a^ **(kg·m**^**-2**^**)**			
*males*	46.0 (44.4, 47.2)	42.5 (38.7, 45.9)	43.3 (41.0, 49.3)
*females*	40.7 (37.3, 46.5)	40.5 (37.8, 45.4)	42.9 (40.5, 46.3)
**Weight (kg)**			
*males*	155.6 (153.5, 163.3)	140.7 (124.0, 151.2)	144.8 (130.9, 155.3)
*females*	117.0 (104.5, 134.6)	116.7 (103.7, 131.8)	119.2 (107.9, 128.1)
**Fat-free mass**^b^^**,**^ ^c^ **(kg)**			
*males*	86.4 (82.2, 93.4)	81.0 (78.6, 89.0)	84.6 (76.0, 88.6)
*females*	60.8 (56.5, 66.9)	60.3 (57.6, 67.8)	62.0 (56.2, 63.6)
**Total fat**^b^^**,**^ ^d^**(kg)**			
*males*	55.0 (52.0, 58.8)	43.2 (35.3, 51.9)	45.6 (38.8, 51.8)
*females*	45.7 (40.0, 52.2)	40.7 (37.3, 58.0)	46.3 (40.5, 53.6)
**Android:gynoid fat ratio**^b^^**,**^ ^e^			
males***	0.74 (0.69, 0.78)	0.85 (0.76, 1.01)	0.97 (0.89, 1.15)
*females*	0.53 (0.50, 0.59)	0.53 (0.47, 0.62)	0.54 (0.47, 0.60)
**Visceral fat**^b^ **(g)**			
males**	563 (542, 628)	960 (831, 1062)	1124 (922, 1493)
*females*	790 (550, 887)	732 (705, 828)	727 (544, 982)
**Waist circumference**^b^ **(cm)**			
*males*	152.0 (550, 887)	144.3 (131.7, 149.5)	147.3 (137.1, 151.6)
*females*	135.6 (124.7, 141.9)	129.4 (125.1, 142.3)	135.7 (132.7, 140.9)
**Hip circumference**^f^ **(cm)**			
males*	135.8 (134.0, 142.0)	123.3 (117.5, 129.8)	129.3 (124.8, 131.3)
*females*	134.0 (126.0, 147.0)	130.0 (125.0, 142.0)	136.0 (130.0, 140.0)

Data presented as median (25th, 75th percentile). Differences between genotype groups determined by Kruskal-Wallis test,

* p<0.05,

** p<0.01,

*** p<0.005. *P*-values are reported with adjustment for ties.

^a^BMI: body mass index.

^b^From DXA.

^c^Fat-free mass: lean mass excluding right and left arms.

^d^Total fat: fat mass excluding right and left arms.

^e^Android:gynoid fat ratio: android fat mass divided by gynoid fat mass.

^f^Measured with measuring tape.

In a dropout analysis comparing the measures presented by sex in Table 1 for the 79 participants who performed the clamp (54 women and 25 men) with the 18 participants who did not perform the clamp (13 women and 5 men), no significant differences were found.

Fifteen participants (6 males, 9 females) smoked daily (median 10 cigarettes/d). There were no differences between males and females in number of cigarettes smoked per day (p = 0.850), nor differences between genotypes in sex-stratified analyses (p = 0.750 for men, and p = 0.833 for females). Regarding participants’ medications, 10% used thyroid replacement hormones, 20% antihypertensive medications, and 16% of the women used hormonal contraceptives (S1 Table). Median (25th, 75th percentile) for variables of glucose tolerance and IS, for males and females, are found in S2 Table.

### Meal test results

**Fig 2:**
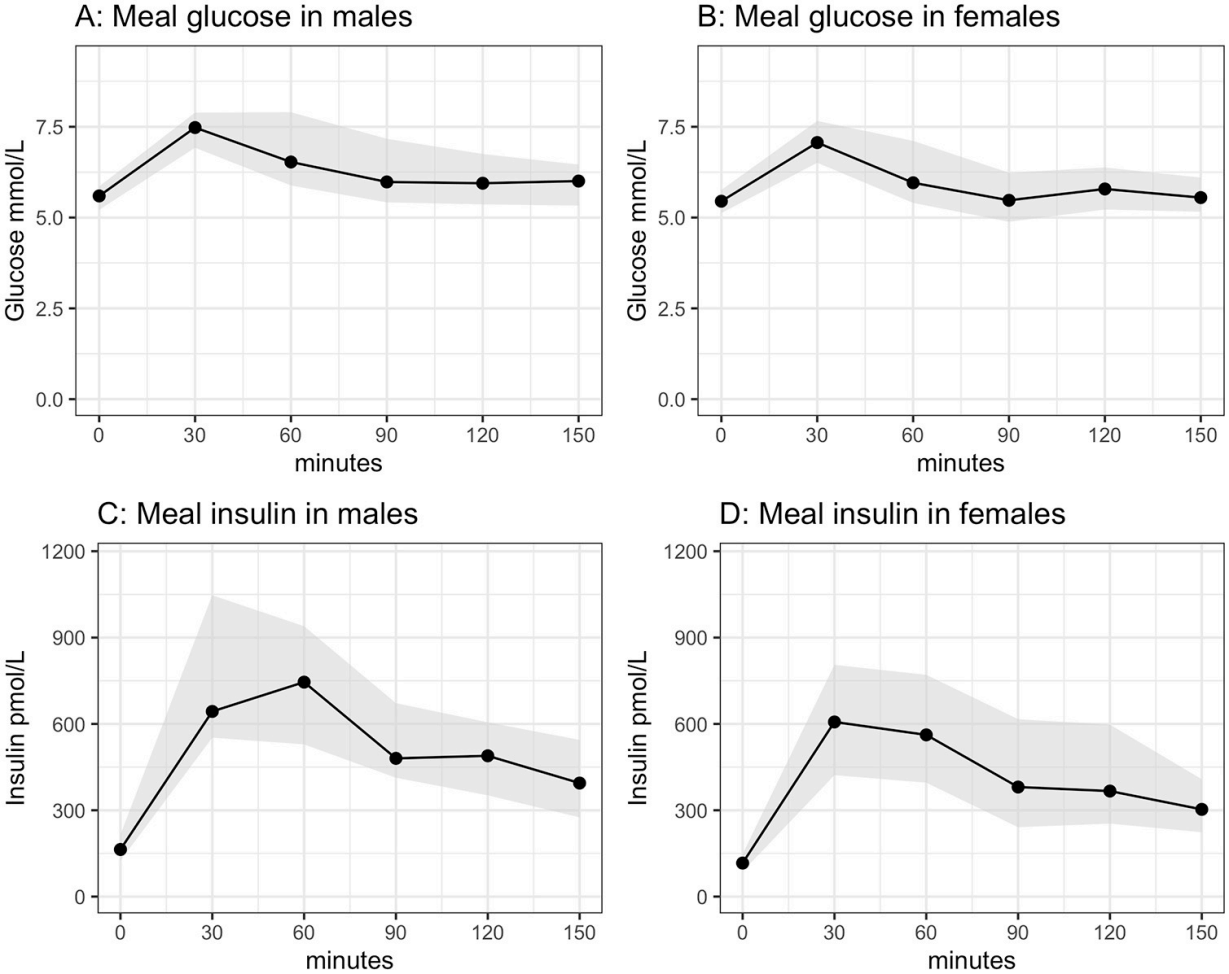
Median fasting and postprandial glucose and insulin levels during meal test. Black circles connected with black solid line show medians, and light grey area shows the area from the 25th (Q1) to the 75th percentile (Q3). Glucose in upper row (A, B) and insulin in the bottom row (C, D), with males in the left columns and females in the right columns.

Postprandial glucose levels peaked at 30 min and remained slightly elevated 150 min after food intake for males and females [Fig 2(A) and 2(B), S2 Table]. A similar pattern was seen for insulin [Fig 2(C) and 2(D), S2 Table].

Genotype effects on glucose tolerance were only seen in males, where genotype A/T was associated with a significantly higher glucose level than genotypes T/T and A/A at 30 min, and also higher levels of glucose at 150 min than genotype T/T (Table 2, S3 Table). There was no genotype effect on insulin responses at 30 min and 150 min (S4 Table) or areas under the glucose and insulin curves for either sex (S5 and S6 Tables).

**Table 2:** Genotype differences in postprandial glucose for time points 30 min and 150 min for males and females as estimated by the LMM analysis.

	Genotype differences in postprandial glucose (mmol/L)		
	A/T—T/T	A/A—A/T	A/A—T/T
	Estimates (99% CI) (mmol·L^-1^)	Estimates (99% CI) (mmol·L^-1^)	Estimates (99% CI) (mmol·L^-1^)
***Males***			
30 min	1.36* (0.42, 2.29)	-0.88* (-1.65, -0.12)	0.48 (-0.46, 1.41)
150 min	1.16* (0.21, 2.10)	-0.37 (-1.14, 0.39)	0.79 (-0.16, 1.73)
***Females***			
30 min	0.25 (-0.20, 0.70)	0.12 (-0.35, 0.58)	0.36 (-0.06, 0.79)
150 min	-0.03 (-0.48, 0.43)	0.14 (-0.33, 0.62)	0.11 (-0.32, 0.55)

* Significant difference between genotypes (99% bootstrap percentile CI does not include 0).

However, males with genotype A/A had a significantly lower IS than males with genotype T/T, showed by a lower value of the Matsuda index (Table 3 and S7 Table). There was no genotype effect on IS as measured in the meal test among females.

**Table 3:** Genotype differences in total insulin sensitivity (Matsuda index) derived from the meal test for males and females as estimated by the RLM analysis.

	Genotype differences in Matsuda index scores		
	A/T—T/T	A/A—A/T	A/A—T/T
	Estimates (99% CI)	Estimates (99% CI)	Estimates (99% CI)
*Males*	-0.65 (-1.69, 0.06)	-0.16 (-0.81, 0.49)	-0.81* (-1.84, -0.09)
*Females*	-0.09 (-1.04, 0.90)	-0.38 (-1.37, 0.63)	-0.47 (-1.36, 0.48)

* Significant difference between genotypes (99% bootstrap BCa CI does not include 0).

### Clamp performance

**Fig 3:**
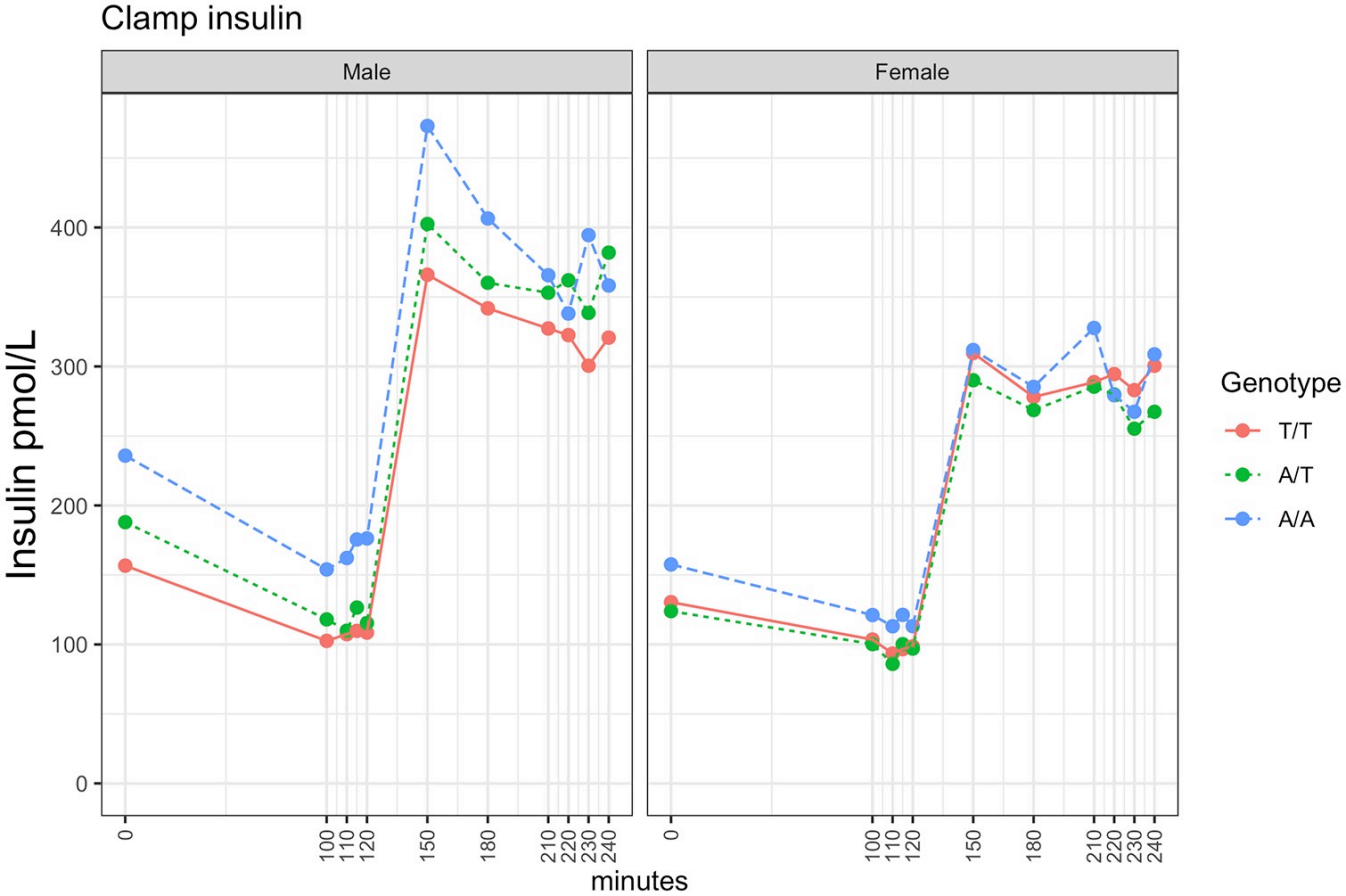
Median serum insulin concentration over time during clamp by sex and genotype. Insulin infusion started at 120 min. Kruskal-Wallis *p*-values for testing for difference in insulin concentration between genotypes at basal time (average of 110, 115 and 120 min) were 0.33 (males) and 0.40 (females) and clamped time (average of 220, 230 and 240 min) were 0.35 (males) and 0.80 (females). Males in left panel, females in right panel.

During the 2 h hyperinsulinemic euglycemic clamp all genotype groups maintained euglycemia. Mean values of glucose were within +/-10% of the target range with values ranging from 3% below to 7% above 4.5 mmol·L^-1^ for each genotype group and for each sex-stratified genotype group (results not shown). There were no statistical differences between genotypes in insulin levels at basal (100–120 min) and at steady state during the end of the clamp (220–240 min), (Fig 3), neither in the reduction in glucagon level from min 100 to 240 (S2 Table).

#### Endogenous glucose production

**Fig 4:**
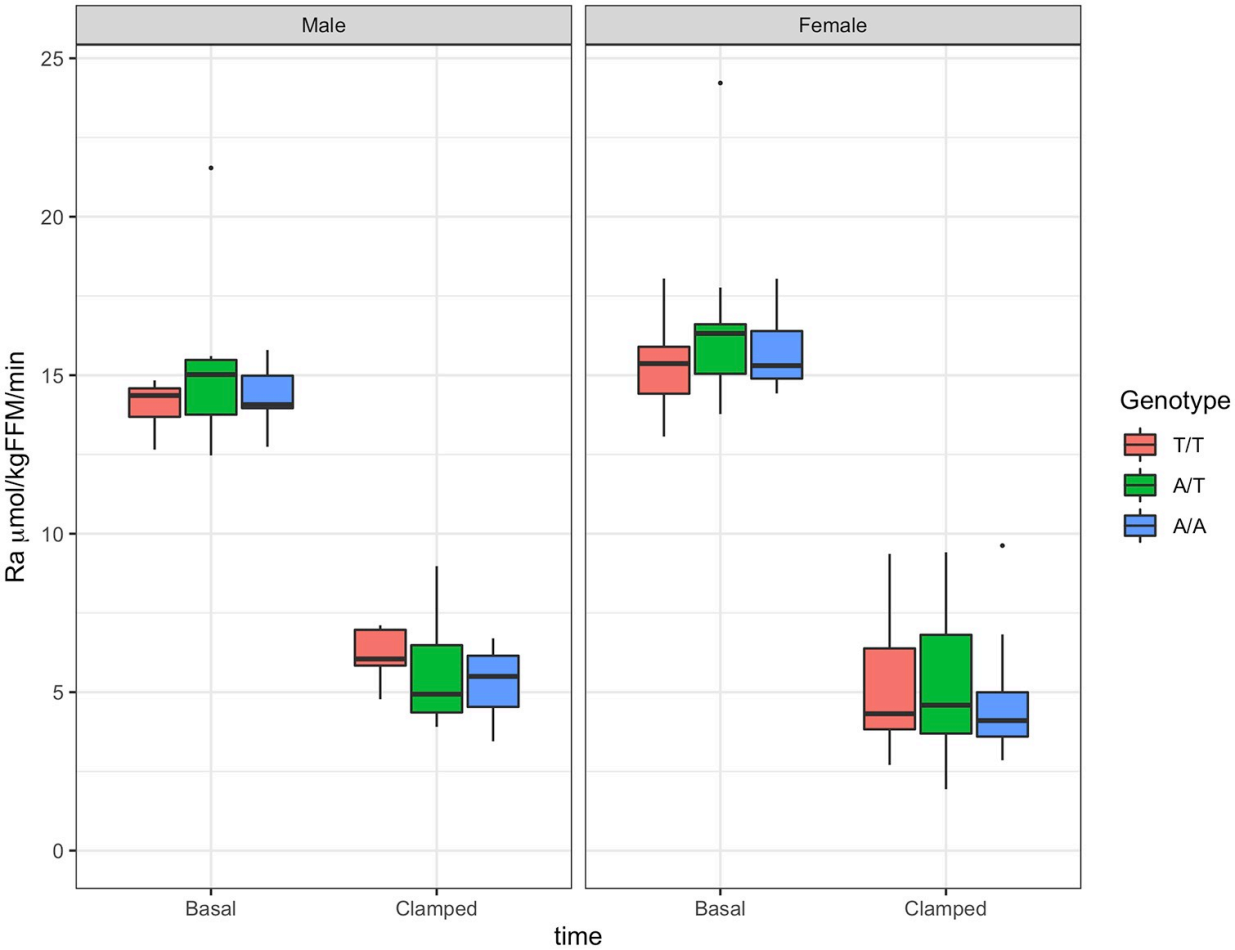
Endogenous glucose production (EGP) by sex and genotype. EGP at basal time and in response to insulin infusion of 0.3 mU·kg^-1^·min^-1^. Boxplots for males in left panel and for females in right panel.

Endogenous glucose production was not affected by genotype for either males or females (Fig 4, S8 Table). It was significantly suppressed at the end of the hyperinsulinemic clamp for all genotypes and both sexes; however, there was no effect by genotype in insulin-suppressed EGP (Table 4, Fig 4, S8 Table). There was no effect of genotype on HIR, in either males or females (S9 Table).

**Table 4:** Time and genotype differences^a^ in EGP, glucose Rd, and MCR, for males and females as estimated by the LMM analysis.

	Genotype differences in EGP, glucose Rd, and MCR		
	A/T—T/T	A/A—A/T	A/A—T/T
	Estimates (99% CI)	Estimates (99% CI)	Estimates (99% CI)
***Males***			
EGP, μmol·kg_FFM_^-1^·min^-1^	-1.69 (-4.73, 1.33)	0.52 (-1.96, 3.00)	-1.18 (-4.25, 1.85)
Glucose Rd, μmol·kg_FFM_^-1^·min^-1^	-2.29 (-5.45, 0.85)	-0.96 (-3.53, 1.66)	-3.25* (-6.44, -0.11)
Glucose MCR, ml·kg_FFM_^-1^·min^-1^	-0.43 (-1.07, 0.21)	-0.26 (-0.78, 0.76)	-0.69 * (-1.34, -0.05)
***Females***			
EGP, μmol·kg_FFM_^-1^·min^-1^	-1.50 (-3.21, 0.22)	0.49 (-1.28, 2.28)	-1.00 (-2.71, 0.71)
Glucose Rd, μmol·kg_FFM_^-1^·min^-1^	-2.29 (-5.45, 0.85)	-0.96 (-3.53, 1.66)	-3.25* (-6.44, -0.11)
Glucose MCR, ml·kg_FFM_^-1^·min^-1^	-0.43 (-1.07, 0.21)	-0.26 (-0.78, 0.76)	-0.69 * (-1.34, -0.05)

^a^The difference in change from basal to clamped time between the genotypes, i.e. the difference in insulin-induced suppression of EGP (glucose Ra), and the difference in insulin-induced increase of glucose Rd and MCR.

* Significant difference between genotypes (99% bootstrap percentile CI does not include 0).

#### Glucose uptake

**Fig 5:**
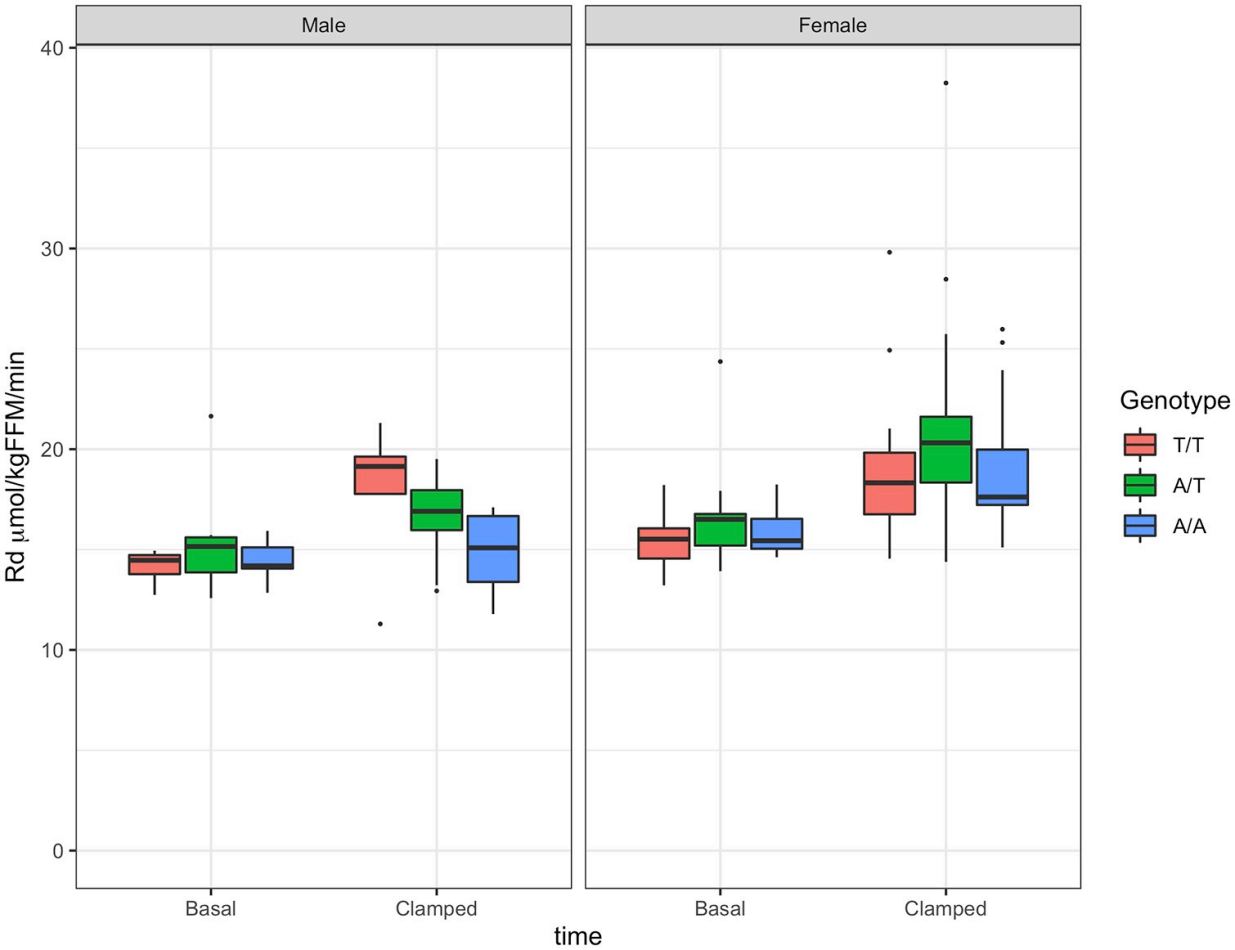
Glucose uptake (Rd) by sex and genotype. Glucose Rd at basal time and in response to insulin infusion (0.3 mU·kg^-1^·min^-1^). Boxplots for males in left panel and for females in right panel.

**Fig 6:**
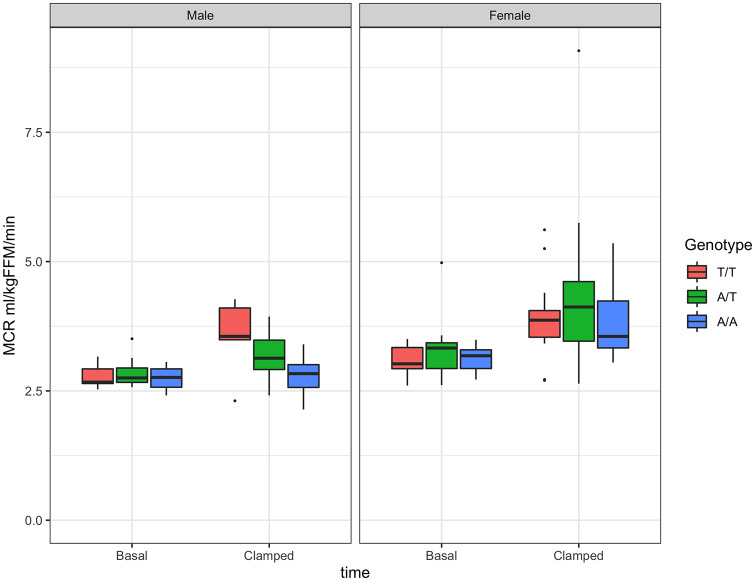
Metabolic clearance rate of glucose (MCR) by sex and genotype. MCR at basal time and in response to insulin infusion (0.3 mU·kg^-1^·min^-1^). Boxplots for males in left panel and for females in right panel.

Glucose uptake increased significantly in females of all genotypes and in males for genotype T/T, as percent increase in glucose Rd from basal (S10 Table). A genotype effect was observed in males where genotype A/A had significantly lower glucose Rd than genotype T/T (Table 4, Fig 5). Results for glucose MCR were, as expected, similar to those of Rd (Table 4, Fig 6, S11 Table).

A significant difference in insulin-induced increase in glucose MCR from basal was observed between males with genotype T/T vs. A/A (Table 4, S7 Table).

There were no significant differences between genotypes in GIR, for either males or females (S12 Table).

#### Sub-group analyses on younger men (< 45 years)

Our results of differences in IS between the T/T and A/A men regardless of age were not negated when we analyzed measures of IS on a subgroup consisting of males <45 years of age. Males with genotype T/T still had a higher score on the Matsuda index than the other two groups of younger males, and lower 30 min and 150 min glucose levels than the A/T group. Insulin stimulated glucose Rd and MCR were significantly lower in young males with genotype A/A than T/T. Further, the visceral fat mass and android:gynoid fat ratio were still lower in the T/T than the A/T and A/A genotype groups (S13 Table).

#### Sex differences in glucose metabolism, independent of genotype

The proportions of participants with genotypes T/T, A/T and A/A did not differ significantly between the sexes (*p*-value 0.182 and *p*-value 0.318, Pearson chi^2^ for meal test participants and clamp participants, respectively), and this allowed for comparisons of meal and clamp responses between males and females (but without adjusting for genotype). Females had significantly better hepatic and total IS and better glucose tolerance than males (details in S14 Table).

## Discussion

This study provides metabolic data that focuses on the genetics of FTO in subjects with comparable and considerable obesity. In designing the study we were aware that obesity *per se* is bound to markedly influence parameters of metabolism and to produce insulin resistance [42]. Therefore, it was important to attain comparable levels of obesity in our study population. Further, to increase the possibility of detecting differences due to genotype we designed the study to contain approximately the same number of heterozygotes and homozygotes for a risk and non-risk allele of FTO. The proportion of females in the study was double that of males and overall females showed better IS and glucose tolerance than males. We wanted to study the metabolic effects of the FTO risk allele without potential disturbances represented by differing biological sex, and therefore we chose to analyze the effects separately for males and females. The data also allowed us to compare differences in glucose metabolism between sexes, independent of genotype.

The liver is essential in glucose homeostasis as a regulator of carbohydrate storage (in the fed state) and supply (in the fasted stage). It is of clinical interest to document or rule out abnormal hepatic sensitivity to insulin because of its linkage to increased risk of T2DM. The FTO gene is strongly expressed in hepatic tissue and FTO genotype may impact on hepatic glucose production, which is the main contributor to EGP in the fasted state [7]. To our knowledge, no previous studies have assessed the association between the FTO risk allele and EGP in a population of healthy individuals with severe obesity. We considered the possibility that parameters of glucose output and uptake may be over interpreted as impaired IS [43] if expressed per kg body weight rather than per kg FFM in individuals with severe obesity, thus possibly affecting on the genotype results. Hence, and in accordance with common practice in the field, we have given results on glucose production and uptake per FFM.

The main result of the current study is that we found no genotype effects on hepatic IS (basal or insulin suppressed) in either sex. However, among males the total IS (clamp glucose Rd and MCR) was lower in the homozygous risk allele carriers (A/A) compared with non-carriers (T/T). The IS results of the meal test (the Matsuda insulin sensitivity index) seemed to be in line with the clamp data. Additionally, the glucose tolerance results of the meal test were affected negatively by one or two risk-alleles, still in males only. Independent of genotype, females showed a better hepatic and total IS than males, and a better glucose tolerance.

We are not aware of other studies on genotype effects of FTO on hepatic IS measured with tracer-technology in healthy individuals with obesity. Therefore, our novel results of no effect of FTO genotype on hepatic IS in subjects with severe obesity is an important contribution to the field. Also, our results of an FTO risk allele effect on total IS in males with severe obesity, should be of clinical interest. The fact that we, with the methods used, confirm known effects by biological sex on IS [44] in our view strengthens the validity of the genotype results we present.

Because total glucose uptake is associated with muscle mass, we considered whether differences in the amount of muscle mass could explain the finding of a lower insulin stimulated glucose uptake among male homozygotes of the risk allele (A/A) compared with homozygotes of the non-risk allele (T/T). However, there were no differences in FFM between those genotype groups. As for differences in fat mass, interestingly, males with genotype A/T and A/A had higher visceral fat mass and higher android:gynoid fat ratio than the T/T genotype group. To account for any age associated differences between the male groups we performed sub-group analyses on the males who were less than 45 y old. There were no differences in age between the three genotype groups in this younger male dataset and the results indicating a better IS among the T/T carriers remained unchanged. This might suggest that in males, carrying two copies of the non-risk variant of the rs9939609 FTO gene gives a metabolically healthier fat mass that is associated with a better total IS [45,46]. We were, however, unable to detect the known association between healthier fat accumulation and hepatic IS [45,46] in our sex-stratified genotype groups. Females had lower visceral fat mass and android:gynoid fat ratio than males, and this could account for the better IS we registered in females than in males (discussed below). Importantly, insulin stimulated glucose uptake was not tested in full given the low insulin dose clamp that was used in current study, and results on insulin stimulated glucose uptake, therefore, should be interpreted with that in mind.

Our results of a negative association between insulin sensitivity and the FTO risk allele in males only, are not in accordance with studies in children and adolescents that find a positive association between insulin resistance and the FTO risk allele mainly in females [18,21]. It has been speculated that these observed sex-differences in the young could be due to increased fat mass in girls and a different fat distribution during childhood compared to boys [18]. Our results among females are perhaps more in line with those reported in a study of female Indonesian adolescents that did not find an effect of FTO rs9939609 on parameters of insulin resistance based on fasting glucose and insulin concentration [19].

Previously, we genotyped and analyzed ~26000 participants in the population based HUNT study, using stratified analysis that allowed us to detect age- and gender-related influences on the genetic impact of FTO [23]. The results from that study indicate that males seem to be more affected by FTO in terms of weight than females; a sex-related finding that is consistent with the present study.

Independent of genotype our female participants displayed better overall IS than the males. In view of previous studies (reviewed in Diabetologia) [44], such a sex difference was perhaps to be expected (although another review points to discrepancies between studies) [47]. Effects of biological sex on EGP is sparsely investigated and results inconclusive [47]. In this context our study provides novel data on EGP that was more profoundly suppressed by insulin in females than in males. Even though our clamp was low-dose hyperinsulinemic and not optimal to measure total IS, our results confirm the reviewed results of better total IS among females compared with males [44]. Moreover, our findings on biological sex appear novel in so far that we have compared metabolic parameters at similar levels of obesity in men and women.

Our clamp data were supplemented by estimates of glucose and insulin responses to an isocaloric real-life meal of 600 kcal that had the same carbohydrate content as an oral glucose tolerance test [25]. For our participants who had severe obesity but were otherwise clinically healthy, it was difficult from meal test data to compare capacities for insulin secretion when blood glucose levels were as expected in subjects without diabetes. Our results are thus in line with those of Goltz et al. [48]. They used a test meal with a higher energy and carbohydrate content than ours, yet did not detect obvious genotype effects from FTO rs9939609 on postprandial glucose and insulin responses.

While most human studies examining the effect of the FTO rs9939609 risk allele on glucose tolerance and IS have assessed only fasting glucose and insulin levels, few have measured postprandial glucose and insulin responses [10,49,50]. To our knowledge, only one study has assessed postprandial blood glucose and insulin responses to a real life-mimicking meal [48], while no studies have assessed the association between the FTO risk allele and EGP *in vivo*. There are strengths and limitations to our study. One of its strengths is that the study population was well characterized. Further, as detailed in the methods section, investigators and participants were blinded to the genotype alleles during the study. Also, we tried to minimize confounding of the IS results by instructing the participants to avoid strenuous physical activity for one day before the meal test and two days before the clamp, and in the evening before the clamp it was arranged for all participants to consume an identical meal. The method of measuring hepatic IS with a low-dose hyperinsulinemic clamp combined with glucose tracer is considered a gold standard method. Moreover, in the statistical analyses we have chosen not to rely on asymptotic distributions of test statistics, and instead used bootstrapping to produce confidence intervals. This is partly due to the statistically rather small sample sizes for each sex, and it is in line with the current trend in research moving away from the strong focus on *p*-values and instead looking at effect sizes through confidence intervals. Finally, we would consider our study to have a unique design and focus. Related to this, as far as we know our study population is still the largest one of individuals with uniform severe obesity in whom glucose metabolism has been extensively investigated with a focus on FTO genetics.

As to limitations, a two-step clamp which includes a succeeding higher infusion rate of insulin would have allowed for an estimation of a near-maximal impact of insulin on glucose uptake and a more in depth assessment of whole body IS. Even so, the combination of low-dose hyperinsulinemic clamp and the use of the glucose tracer made it possible to give estimates of Rd and MCR as an assessment of total IS. Because a high-dose hyperinsulinemic clamp is closer to the gold standard for measuring total IS, our results have to be cautiously interpreted. Another limitation is the composition of the study population. The recruitment was based on referrals for treatment to our hospital and the preponderance of females vs. males likely reflects that the bulk of referrals were for females. This in turn could possibly reflect that a greater proportion of women than men in Norway have obesity grades 2 or 3 [51] and that women are reported to visit primary physicians and specialists more than men [52]. Notwithstanding a common phenotype of obesity there was heterogeneity between our subjects in other respects, for instance the insulin responses during the meal test and the GIR during the clamps, as well as the lowering effects on EGP. This variability could potentially occlude modest but real effects by genotype. A random sample from a population-based study would have allowed more general conclusions from our results. Finally, we acknowledge that the advanced statistics used here only partly remedy for a non-optimal size of the study population, and a larger study population would have strengthened the validity of our findings.

### Conclusion

To conclude, having used the gold standard method for measuring EGP we did not find an effect on hepatic IS by the FTO gene, but a risk allele effect on total IS in males. The new knowledge was gained in a setting of universal obesity and taking into account modifying influences by biological sex. Our findings extend the current knowledge on the impact of the FTO gene on intermediary metabolism but should be verified in future studies with larger samples.

## Supporting information

S1 Table Prescription medications used by the 97 participants. (DOCX)

S2 TableMedian glucose tolerance and insulin sensitivity variables for males and females by genotype.Data presented as median (25th, 75th percentile). ^a^Matsuda index: $\text{Index}\;\text{of}\;\text{whole}\;\text{body}\;\text{insulin}\;\text{sensitivity}=\frac{10000}{\sqrt{\left(\text{fasting}\;\text{glucose}\;\text{conc}\cdot \text{fasting}\;\text{insulin}\;\text{conc})\cdot (\text{mean}\;\text{glucose}\;\text{conc}\cdot \text{mean}\;\text{insulin}\;\text{conc}\right)}}$ ^b^FFM: Fat-free mass is lean mass excluding right and left arms measured by DXA. ^c^HIR: Hepatic insulin resistance index = basal glucose Ra·basal insulin. ^d^Kruskal-Wallis test for differences in delta glucagon levels between genotype groups, *p* = 0.523 for males and *p* = 0.263 for females.(DOCX)

S3 Table Parameter estimates and contrasts for combinations of time (30 and 150 minutes) and genotype for each sex for the meal test glucose analyses (mmol/L), with 99% bootstrap percentile CI. CI confidence interval. Intraclass correlation estimates were 0.57 (males) and 0.54 (females). * Significant difference between genotypes (99% bootstrap percentile CI does not include 0).
(DOCX)

S4 Table Parameter estimates and contrasts for combinations of time (30 and 150 minutes) and genotype for each sex for the meal test insulin analyses (pmol/L), with 99% bootstrap percentile CI. CI confidence interval. Intraclass correlation estimates were 0.27 (males) and 0.46 (females).
(DOCX)

S5 Table Meal test glucose AUC minutes 1–150, with 99% bootstrap BCa CI. AUC: Area under curve. BCa CI: Bias-corrected and accelerated bootstrap intervals.
(DOCX)

S6 Table Meal test insulin AUC minutes 0–150, with 99% bootstrap BCa CI. AUC: Area under curve. BCa CI: Bias-corrected and accelerated bootstrap intervals.
(DOCX)

S7 TableParameter estimates and contrasts for combinations of genotype for each sex for the meal test Matsuda index, with 99% bootstrap BCa CI.Matsuda index: $\text{Index}\;\text{of}\;\text{whole}\;\text{body}\;\text{insulin}\;\text{sensitivity}=\frac{10000}{\sqrt{\left(\text{fasting}\;\text{glucose}\;\text{conc}\cdot \text{fasting}\;\text{insulin}\;\text{conc})\cdot (\text{mean}\;\text{glucose}\;\text{conc}\cdot \text{mean}\;\text{insulin}\;\text{conc}\right)}}$ BCa CI: Bias-corrected and accelerated bootstrap intervals. * Significant difference between genotypes (99% bootstrap percentile CI does not include 0).(DOCX)

S8 Table Parameter estimates and contrasts of time and genotype for each sex for the LMM endogenous glucose production (EGP) analyses (μmol/·kg_FFM_/min), with 99% bootstrap percentile CI. LMM: Linear mixed effects model; CI confidence interval. Intraclass correlation estimates were 0.03 (males) and 0.44 (females).
(DOCX)

S9 Table Parameter estimates and contrasts for combinations of genotype for each sex for the Hepatic insulin resistance index (HIR), with 99% bootstrap BCa CI. BCa CI: Bias-corrected and accelerated bootstrap intervals.
(DOCX)

S10 Table Parameter estimates and contrasts of time and genotype for each sex for the LMM glucose Rd analyses (μmol/kg_FFM_/min), with 99% bootstrap percentile CI. LMM: Linear mixed effects model; CI confidence interval. Intraclass correlation estimates were 0.46 (males) and 0.19 (females). * Significant difference between genotypes (99% bootstrap percentile CI does not include 0).
(DOCX)

S11 Table Parameter estimates and contrasts of time and genotype for each sex for the LMM glucose MCR analyses (ml/kg_FFM_/min), with 99% bootstrap percentile CI. LMM: Linear mixed effects model; CI confidence interval. Intraclass correlation estimates were 0.34 (males) and 0.18 (females). * Significant difference between genotypes (99% bootstrap percentile CI does not include 0).
(DOCX)

S12 Table Parameter estimates and contrasts for combinations of genotype for each sex for glucose infusion rate (GIR) (μmol/kg_FFM_/min), with 99% bootstrap BCa CI. BCa CI: Bias-corrected and accelerated bootstrap intervals.
(DOCX)

S13 Table Anthropometric and body composition measures by genotype group for men younger than 45 years (*n* = 19). Data presented as median (25th, 75th percentile). Differences between genotype groups determined using Kruskal-Wallis test, * *p* <0.05, ** p<0.01. *P*-values are reported with adjustment for ties. ^a^BMI: Body mass index; ^b^From DXA; ^c^Fat-free mass: Lean mass excluding right and left arms; ^d^Total fat: Fat mass excluding right and left arms; ^e^Android:gynoid fat ratio: Android fat mass/gynoid fat mass; ^f^Measured with measuring tape.
(DOCX)

S14 TableMedian for glucose tolerance and insulin sensitivity variables for males and females independent of genotype.Values are median (25th, 75th percentile). ^a^*P*-values derived from two-sample Wilcoxon rank-sum (Mann-Whitney) test for differences between males and females. ^b^Matsuda index: $\text{Index}\;\text{of}\;\text{whole}\;\text{body}\;\text{insulin}\;\text{sensitivity}=\frac{10000}{\sqrt{\left(\text{fasting}\;\text{glucose}\;\text{conc}\cdot \text{fasting}\;\text{insulin}\;\text{conc})\cdot (\text{mean}\;\text{glucose}\;\text{conc}\cdot \text{mean}\;\text{insulin}\;\text{conc}\right)}}$ ^c^FFM: Fat-free mass is lean mass excluding right and left arms measured by DXA. ^d^HIR: Hepatic insulin resistance index = basal glucose Ra · basal insulin.(DOCX)
