# Supplementary material for: The fat mass and obesity-associated (FTO) gene allele rs9939609 and glucose tolerance, hepatic and total insulin sensitivity, in adults with obesity
Source: PLoS One. 2021 Mar 8;16(3):e0248247. doi: 10.1371/journal.pone.0248247 (PMC7939351; doi:10.1371/journal.pone.0248247)
Supplement: S1 Table — (DOCX) [file pone.0248247.s001.docx]

**S1 Table. Prescription medications used by the 97 participants.**

| **Medication** | **T/T**  *n*=32 (26 females) | **A/T**  *n*=31 (19 females) | **A/A**  *n*=34 (22 females) |
| --- | --- | --- | --- |
| Hormonal contraceptive (women only) | 2 (7.7%) | 4 (21.1%) | 5 (22.7%) |
| Thyroid replacement hormones | 5 (15.6%) | 4 (12.9%) | 1 (2.9%) |
| Lipid modifying | 1 (3.1%) | 3 (9.7%) | 0 (0%) |
| Antihypertensive | 4 (12.5%) | 6 (19.4%) | 9 (26.5%) |
| Diuretic | 2 (6.3%) | 2 (6.5%) | 1 (2.9%) |
| Antipsychotic | 1 (3.1%) | 1 (3.2%) | 1 (2.9%) |
| Weight-loss inducing | 1 (3.1%) | 0 (0%) | 1 (2.9%) |
