# Supplementary material for: The fat mass and obesity-associated (FTO) gene allele rs9939609 and glucose tolerance, hepatic and total insulin sensitivity, in adults with obesity
Source: PLoS One. 2021 Mar 8;16(3):e0248247. doi: 10.1371/journal.pone.0248247 (PMC7939351; doi:10.1371/journal.pone.0248247)
Supplement: S2 Table — Data presented as median (25th, 75th percentile). aMatsuda index: Indexofwholebodyinsulinsensitivity=10000(fastingglucoseconc⋅fastinginsulinconc)⋅(meanglucoseconc⋅meaninsulinconc) bFFM: Fat-free mass is lean mass excluding right and left arms measured by DXA. cHIR: Hepatic insulin resistance index = basal glucose Ra·basal insulin. dKruskal-Wallis test for differences in delta glucagon levels between genotype groups, p = 0.523 for males and p = 0.263 for females. (DOCX) [file pone.0248247.s002.docx]

**S2 Table. Median glucose tolerance and insulin sensitivity variables for males and females by genotype.**

|  | **T/T** | **A/T** | **A/A** |
| --- | --- | --- | --- |
| **Meal test, males** | *n*=6 | *n*=12 | *n*=12 |
| Glucose 0min (mmol·L^-1^) | 5.14 (4.95, 5.4) | 5.86 (5.5, 6.32) | 5.60 (5.3, 5.69) |
| Glucose 30min (mmol·L^-1^) | 6.66 (6.56, 6.95) | 7.7 (7.22, 9.54) | 7.55 (6.89, 7.90) |
| Glucose 150min (mmol·L^-1^) | 5.31 (5.06, 5.79) | 6.16 (5.46, 6.60) | 6.09 (5.81, 6.62) |
| Insulin 0min (pmol·L^-1^) | 147.16 (139.2, 171.90) | 204.36 (145.36, 252.83) | 227.42 (159.89, 262.91) |
| Insulin 30min (pmol·L^-1^) | 692.55 (638.72, 701.64) | 819.85 (611.87, 1145.26) | 996.54 (629.52, 1232.99) |
| Insulin 150min (pmol·L^-1^) | 413.55 (249.61, 461.75) | 401.26 (306.21, 515.66) | 646.16 (398.71, 727.72) |
| Matsuda insulin sensitivity index^a^ | 2.64 (2.05, 2.78) | 1.86 (1.62, 2.05) | 1.59 (1.29, 2.22) |
| **Clamp, males** | *n*=5 | *n*=10 | *n*=10 |
| EGP basal (µmol·kg_FFM_^-1^·min^-1^)^b^ | 14.36 (13.69, 14.59) | 15.02 (13.54, 15.51) | 14.07 (13.96, 15.07) |
| EGP clamped (µmol·kg_FFM_·min^-1^)^b^ | 6.05 (5.84, 6.97) | 4.94 (4.29, 6.67) | 5.50 (4.52, 6.17) |
| EGP % decrease | 55.8 (51.5, 60.7) | 68.0 (57.3, 69.9) | 61.7 (55.9, 70.9) |
| Glucose Rd basal $(\mu$mol·kg_FFM_^-1^·min^-1^) ^b^ | 14.47 (13.78, 14.72) | 15.15 (13.65, 15.64) | 14.19 (14.05, 15.20) |
| Glucose Rd clamped $(\mu$mol·kg_FFM_·min^-1^)^b^ | 19.14 (17.77, 19.63) | 16.91 (15.92, 18.06) | 15.09 (13.04, 17.05) |
| Glucose Rd % increase | 30.0 (29.0, 31.3) | 3.3 (2.7, 21.8) | 6.6 (-5.1, 9.7) |
| Glucose MCR basal (ml·kg_FFM_·min^-1^)^b^ | 2.67 (2.64, 2.93) | 2.75 (2.66, 2.98) | 2.76 (2.56, 2.96) |
| Glucose MCR clamped (ml·kg_FFM_·min^-1^)^b^ | 3.55 (3.49, 4.10) | 3.13 (2.90, 3.50) | 2.83 (2.54, 3.03) |
| Glucose MCR % increase | 33.1 (29.7, 37.9) | 7.3 (1.2,23.0) | 3.4 (-2.4, 10.5) |
| GIR (µmol·kg_FFM_·min^-1^)^b^ | 10.97 (9.92, 12.30) | 10.09 (7.50, 11.83) | 7.66 (7.01, 9.84) |
| HIR^c^ | 1578.53 (1254.62, 1657.39) | 1859.99 (1396.96, 2833.53) | 2388.33 (1445.23, 3265.31) |
| Glucagon 100 min (ng·L^-1^) | 76.44 (76.07, 91.31) | 94.51 (77.34, 121.53) | 90.25 (69.40, 105.11) |
| Glucagon 240 min (ng·L^-1^) | 79.48 (64.96, 102.10) | 100.72 (71.95, 105.89) | 89.80 (78.75, 103. 64) |
| $\Delta$ Glucagon (ng·L^-1^)^d^ | -6.33 (-28.30, 6.4) | -16.55 (-17.94, -4.77) | -2.88 (-11.51, 1.79) |
| **Meal test, females** | *n*=26 | *n*=19 | *n*=22 |
| Glucose 0min (mmol·L^-1^) | 5.35 (5.06, 5.60) | 5.34 (5.14, 5.99) | 5.57 (5.30, 6.02) |
| Glucose 30min (mmol·L^-1^) | 6.98 (6.30, 7.45) | 7.05 (6.30, 7.99) | 7.21 (6.51, 7.68) |
| Glucose 150min (mmol·L^-1^) | 5.43 (5.12, 6.04) | 5.68 (5.19, 6.10) | 5.70 (5.19, 6.34) |
| Insulin 0min (pmol·L^-1^) | 120.84 (97.58, 175.45) | 118.31 (99.71, 164.14) | 152.63 (112.13, 176.72) |
| Insulin 30min (pmol·L^-1^) | 725.75 (529.45, 1089.95) | 751.57 (463.21, 880.16) | 685.45 (473.57, 893.26) |
| Insulin 150min (pmol·L^-1^) | 342.11 (214.67, 478.04) | 336.00 (278.04, 522.72) | 403.12 (287.85, 456.52) |
| Matsuda insulin sensitivity index^a^ | 3.20 (1.82, 3.99) | 2.65 (2.22, 3.76) | 2.27 (1.99, 3.01) |
| **Clamp, females** | *n*=25 | *n*=17 | *n*=17 |
| EGP basal (µmol·kg_FFM_^-1^·min^-1^)^b^ | 15.37 (14.34, 15.91) | 16.32 (15.05, 16.61) | 15.30 (14.89, 16.39) |
| EGP clamped (µmol·kg_FFM_·min^-1^)^b^ | 4.32 (3.72, 6.47) | 4.40 (3.40, 6.60) | 4.10 (3.60, 5.00) |
| EGP % decrease | 71.0 (56.0, 75.6) | 71.2 (61.1, 77.8) | 74.7 (67.5, 77.5) |
| Glucose Rd basal $(\mu$mol·kg_FFM_^-1^·min^-1^) ^b^ | 15.52 (14.47, 16.07) | 16.50 (15.20, 16.77) | 15.4415.04, 16.53) |
| Glucose Rd clamped $(\mu$mol·kg_FFM_·min^-1^)^b^ | 18.32 (16.44, 19.91) | 20.32 (18.34, 21.61) | 17.62 (17.22, 19.98) |
| Glucose Rd % increase | 16.7 (10.9, 29.5) | 22.2 (13.1, 30.4) | 16.0 (6.2, 26.7) |
| Glucose MCR basal (ml·kg_FFM_·min^-1^)^b^ | 3.02 (2.93, 3.34) | 3.32 (2.93, 3.43) | 3.18 (2.93, 3.29) |
| Glucose MCR clamped (ml·kg_FFM_·min^-1^)^b^ | 3.87 (3.52, 4.10) | 4.12 (3.46, 4.61) | 3.55 (3.33, 4.24) |
| Glucose MCR % increase | 22.7 (11.1, 34.5) | 18.0 (12.6, 31.9) | 18.8 (5.0, 23.9) |
| GIR (µmol·kg_FFM_·min^-1^)^b^ | 11.63 (8.35, 15.50) | 14.02 (12.30, 17.52) | 13.18 (10.90, 16.69) |
| HIR^c^ | 1520.22 (1146.72, 1876.89) | 1558.17 (1143.91, 2181.57) | 1861.98 (1572.82, 2106.38) |
| Glucagon 100 min (ng·L^-1^) | 69.80 (57.33, 77.04) | 81.80 (62.21, 86.20) | 71.58 (56.65, 81.62) |
| Glucagon 240 min (ng·L^-1^) | 64.30 (56.30, 77.88) | 68.35 (59.56, 77.18) | 59.85 (54.16, 78.80) |
| $\Delta$ Glucagon (ng·L^-1^)^d^ | -2.19 (-14.89, 7.21) | -8.85 (-14.98, -1.05) | -8.41 (-18.35, 2.11) |

Data presented as median (25th, 75th percentile).

^a^Matsuda index: Index of whole body insulin sensitivity

$$=\frac{10000}{\sqrt{(\text{fasting glucose conc}\cdot\text{fasting insulin conc})\cdot(\text{mean glucose conc}\cdot\text{mean insulin conc})}}$$

^b^FFM: Fat free mass is lean mass excluding right and left arms measured by DXA.

^c^HIR: Hepatic insulin resistance index = basal glucose Ra·basal insulin

^d^Kruskal-Wallis test for differences in delta glucagon levels between genotype groups, *p*=0.523 for males and *p*=0.263 for females
