# Supplementary material for: The fat mass and obesity-associated (FTO) gene allele rs9939609 and glucose tolerance, hepatic and total insulin sensitivity, in adults with obesity
Source: PLoS One. 2021 Mar 8;16(3):e0248247. doi: 10.1371/journal.pone.0248247 (PMC7939351; doi:10.1371/journal.pone.0248247)
Supplement: S3 Table — CI confidence interval. Intraclass correlation estimates were 0.57 (males) and 0.54 (females). * Significant difference between genotypes (99% bootstrap percentile CI does not include 0). (DOCX) [file pone.0248247.s003.docx]

**S3 Table.** **Parameter estimates and contrasts for combinations of time (30 and 150 minutes) and genotype for each sex for the meal test glucose analyses (mmol/L), with 99% bootstrap percentile CI.**

|  |  | **Male** (*n*=30) | | | **Female** (*n*=67) | | |
| --- | --- | --- | --- | --- | --- | --- | --- |
| **Genotype** | Time | Estimate | CI Lower | CI Higher | Estimate | CI Lower | CI Higher |
| A/T-T/T | 30 | 1.36* | 0.42 | 2.29 | 0.25 | -0.20 | 0.70 |
| A/A-A/T | 30 | -0.88* | -1.64 | -0.12 | 0.12 | -0.35 | 0.58 |
| A/A-T/T | 30 | 0.48 | -0.46 | 1.41 | 0.36 | -0.06 | 0.79 |
| A/T-T/T | 150 | 1.16* | 0.21 | 2.10 | -0.03 | -0.48 | 0.43 |
| A/A-A/T | 150 | -0.37 | -1.14 | 0.39 | 0.14 | -0.33 | 0.62 |
| A/A-T/T | 150 | 0.79 | -0.16 | 1.73 | 0.11 | -0.32 | 0.55 |
| A/T-T/T | 150-30 | -0.20 | -1.51 | 1.11 | -0.28 | -0.91 | 0.38 |
| A/A-A/T | 150-30 | 0.51 | -0.57 | 1.60 | 0.02 | -0.65 | 0.69 |
| A/A-T/T | 150-30 | 0.31 | -1.00 | 1.63 | -0.26 | -0.86 | 0.35 |

CI confidence interval. Intraclass correlation estimates were 0.57 (males) and 0.54 (females).

* Significant difference between genotypes (99% bootstrap percentile CI does not include 0).
