# Supplementary material for: The fat mass and obesity-associated (FTO) gene allele rs9939609 and glucose tolerance, hepatic and total insulin sensitivity, in adults with obesity
Source: PLoS One. 2021 Mar 8;16(3):e0248247. doi: 10.1371/journal.pone.0248247 (PMC7939351; doi:10.1371/journal.pone.0248247)
Supplement: S4 Table — CI confidence interval. Intraclass correlation estimates were 0.27 (males) and 0.46 (females). (DOCX) [file pone.0248247.s004.docx]

**S4 Table.** **Parameter estimates and contrasts for combinations of time (30 and 150 minutes) and genotype for each sex for the meal test insulin analyses (pmol/L), with 99% bootstrap percentile CI.**

|  |  | **Male** (*n*=30) | | | **Female** (*n*=67) | | |
| --- | --- | --- | --- | --- | --- | --- | --- |
| **Genotype** | Time | Estimate | CI Lower | CI Higher | Estimate | CI Lower | CI Higher |
| A/T-T/T | 30 | 54.25 | -248.78 | 355.25 | -77.53 | -234.16 | 80.92 |
| A/A-A/T | 30 | 52.05 | -193.66 | 298.16 | -2.75 | -166.29 | 159.54 |
| A/A-T/T | 30 | 106.30 | -196.06 | 406.27 | -80.28 | -229.44 | 70.02 |
| A/T-T/T | 150 | 32.98 | -273.13 | 336.04 | -29.79 | -189.61 | 129.39 |
| A/A-A/T | 150 | 165.70 | -81.14 | 412.33 | -12.21 | -176.20 | 160.15 |
| A/A-T/T | 150 | 198.68 | -107.00 | 502.49 | -42.00 | -190.78 | 113.90 |
| A/T-T/T | 150-30 | -21.26 | -446.55 | 400.61 | 47.74 | -177.72 | 274.26 |
| A/A-A/T | 150-30 | 113.65 | -236.21 | 466.19 | -9.45 | -244.38 | 229.42 |
| A/A-T/T | 150-30 | 92.38 | -332.87 | 516.62 | 38.29 | -173.45 | 253.01 |

CI confidence interval. Intraclass correlation estimates were 0.27 (males) and 0.46 (females).
