# Supplementary material for: The fat mass and obesity-associated (FTO) gene allele rs9939609 and glucose tolerance, hepatic and total insulin sensitivity, in adults with obesity
Source: PLoS One. 2021 Mar 8;16(3):e0248247. doi: 10.1371/journal.pone.0248247 (PMC7939351; doi:10.1371/journal.pone.0248247)
Supplement: S5 Table — AUC: Area under curve. BCa CI: Bias-corrected and accelerated bootstrap intervals. (DOCX) [file pone.0248247.s005.docx]

**S5 Table. Meal test glucose AUC minutes 1-150, with 99% bootstrap BCa CI.**

|  | **Male** (*n*=30) | | | **Female** (*n*=67) | | |
| --- | --- | --- | --- | --- | --- | --- |
| **Genotype** | Estimate | CI Lower | CI Higher | Estimate | CI Lower | CI Higher |
| T/T | 877 | 779 | 1374 | 891 | 844 | 983 |
| A/T | 1000 | 926 | 1254 | 899 | 840 | 1010 |
| A/A | 974 | 901 | 1268 | 928 | 876 | 1029 |
| A/T-T/T | 123 | -151 | 288 | 8 | -94 | 107 |
| A/A-A/T | -26 | -192 | 125 | 29 | -75 | 132 |
| A/A-T/T | 98 | -178 | 262 | 37 | -56 | 133 |

AUC: Area under curve.

BCa CI: Bias-corrected and accelerated bootstrap intervals
