# Supplementary material for: The fat mass and obesity-associated (FTO) gene allele rs9939609 and glucose tolerance, hepatic and total insulin sensitivity, in adults with obesity
Source: PLoS One. 2021 Mar 8;16(3):e0248247. doi: 10.1371/journal.pone.0248247 (PMC7939351; doi:10.1371/journal.pone.0248247)
Supplement: S6 Table — AUC: Area under curve. BCa CI: Bias-corrected and accelerated bootstrap intervals. (DOCX) [file pone.0248247.s006.docx]

**S6 Table. Meal test insulin AUC minutes 0-150, with 99% bootstrap BCa CI.**

|  | **Male** (*n*=30) | | | **Female** (*n*=67) | | |
| --- | --- | --- | --- | --- | --- | --- |
| **Genotype** | Estimate | CI Lower | CI Higher | Estimate | CI Lower | CI Higher |
| T/T | 71716 | 44071 | 111891 | 68901 | 57754 | 93677 |
| A/T | 77159 | 57884 | 103780 | 66348 | 52794 | 97259 |
| A/A | 102581 | 83579 | 131076 | 72851 | 60626 | 101450 |
| A/T-T/T | 5444 | -38540 | 40391 | -2554 | -26543 | 21750 |
| A/A-A/T | 25422 | -7424 | 55792 | 6504 | -18709 | 31523 |
| A/A-T/T | 30865 | -11570 | 67390 | 3950 | -18983 | 27433 |

AUC: Area under curve.

BCa CI: Bias-corrected and accelerated bootstrap intervals
