# Supplementary material for: The fat mass and obesity-associated (FTO) gene allele rs9939609 and glucose tolerance, hepatic and total insulin sensitivity, in adults with obesity
Source: PLoS One. 2021 Mar 8;16(3):e0248247. doi: 10.1371/journal.pone.0248247 (PMC7939351; doi:10.1371/journal.pone.0248247)
Supplement: S7 Table — Matsuda index: Indexofwholebodyinsulinsensitivity=10000(fastingglucoseconc⋅fastinginsulinconc)⋅(meanglucoseconc⋅meaninsulinconc) BCa CI: Bias-corrected and accelerated bootstrap intervals. * Significant difference between genotypes (99% bootstrap percentile CI does not include 0). (DOCX) [file pone.0248247.s007.docx]

**S7 Table.** **Parameter estimates and contrasts for combinations of genotype for each sex for the meal test Matsuda index, with 99% bootstrap BCa CI.**

|  | **Male** (*n*=30) | | | **Female** (*n*=67) | | |
| --- | --- | --- | --- | --- | --- | --- |
| **Genotype** | Estimate | CI Lower | CI Higher | Estimate | CI Lower | CI Higher |
| T/T | 2.53 | 2.02 | 3.74 | 2.98 | 2.61 | 4.16 |
| A/T | 1.88 | 1.53 | 2.60 | 2.89 | 2.43 | 4.08 |
| A/A | 1.72 | 1.38 | 2.40 | 2.51 | 2.09 | 3.61 |
| A/T-T/T | -0.65 | -1.69 | 0.06 | -0.09 | -1.04 | 0.90 |
| A/A-A/T | -0.16 | -0.81 | 0.49 | -0.38 | -1.37 | 0.63 |
| A/A-T/T | -0.81* | -1.84 | -0.09 | -0.47 | -1.36 | 0.48 |

Matsuda index: Index of whole body insulin sensitivity

$$=\frac{10000}{\sqrt{(\text{fasting glucose conc}\cdot\text{fasting insulin conc})\cdot(\text{mean glucose conc}\cdot\text{mean insulin conc})}}$$

BCa CI: Bias-corrected and accelerated bootstrap intervals

* Significant difference between genotypes (99% bootstrap percentile CI does not include 0).
