# Supplementary material for: The fat mass and obesity-associated (FTO) gene allele rs9939609 and glucose tolerance, hepatic and total insulin sensitivity, in adults with obesity
Source: PLoS One. 2021 Mar 8;16(3):e0248247. doi: 10.1371/journal.pone.0248247 (PMC7939351; doi:10.1371/journal.pone.0248247)
Supplement: S8 Table — LMM: Linear mixed effects model; CI confidence interval. Intraclass correlation estimates were 0.03 (males) and 0.44 (females). (DOCX) [file pone.0248247.s008.docx]

**S8 Table.** **Parameter estimates and contrasts of time and genotype for each sex for the LMM endogenous glucose production (EGP) analyses (**$\boldsymbol{\mu}$**mol/·kg_FFM_/min), with 99% bootstrap percentile CI.**

|  |  | **Male** (*n*=30) | | | **Female** (*n*=67) | | |
| --- | --- | --- | --- | --- | --- | --- | --- |
| **Genotype** | Time | Estimate | CI Lower | CI Higher | Estimate | CI Lower | CI Higher |
| T/T | % change clamped-basal | -0.56 | -0.69 | -0.41 | -0.66 | -0.72 | -0.60 |
| A/T | % change clamped-basal | -0.63 | -0.72 | -0.54 | -0.71 | -0.77 | -0.65 |
| A/A | % change clamped-basal | -0.63 | -0.72 | -0.54 | -0.71 | -0.76 | -0.65 |
| A/T-T/T | basal | 1.12 | -1.02 | 3.25 | 1.06 | -0.15 | 2.25 |
| A/A-A/T | basal | -0.80 | -2.57 | 0.94 | -0.61 | -1.88 | 0.65 |
| A/A-T/T | basal | 0.31 | -1.81 | 2.46 | 0.45 | -0.76 | 1.66 |
| A/T-T/T | clamped | -0.57 | -2.75 | 1.58 | -0.43 | -1.67 | 0.78 |
| A/A-A/T | clamped | -0.29 | -2.03 | 1.47 | -0.12 | -1.36 | 1.16 |
| A/A-T/T | clamped | -0.86 | -2.99 | 1.29 | -0.55 | -1.74 | 0.68 |
| A/T-T/T | clamped-basal | -1.69 | -4.73 | 1.33 | -1.50 | -3.21 | 0.22 |
| A/A-A/T | clamped-basal | 0.52 | -1.96 | 3.04 | 0.49 | -1.28 | 2.28 |
| A/A-T/T | clamped-basal | -1.18 | -4.25 | 1.85 | -1.00 | -2.71 | 0.71 |

LMM: Linear mixed effects model; CI confidence interval. Intraclass correlation estimates were 0.03 (males) and 0.44 (females).
