# Supplementary material for: The fat mass and obesity-associated (FTO) gene allele rs9939609 and glucose tolerance, hepatic and total insulin sensitivity, in adults with obesity
Source: PLoS One. 2021 Mar 8;16(3):e0248247. doi: 10.1371/journal.pone.0248247 (PMC7939351; doi:10.1371/journal.pone.0248247)
Supplement: S9 Table — BCa CI: Bias-corrected and accelerated bootstrap intervals. (DOCX) [file pone.0248247.s009.docx]

**S9 Table.** **Parameter estimates and contrasts for combinations of genotype for each sex for the Hepatic insulin resistance index (HIR), with 99% bootstrap BCa CI.**

|  | **Male** (*n*=30) | | | **Female** (*n*=67) | | |
| --- | --- | --- | --- | --- | --- | --- |
| **Genotype** | Estimate | CI Lower | CI Higher | Estimate | CI Lower | CI Higher |
| T/T | 1492 | 592 | 2617 | 1541 | 1306 | 2194 |
| A/T | 2057 | 1375 | 2880 | 1685 | 1422 | 2379 |
| A/A | 2388 | 1692 | 3210 | 1852 | 1588 | 2691 |
| A/T-T/T | 565 | -801 | 1800 | 143 | -408 | 708 |
| A/A-A/T | 331 | -692 | 1539 | 167 | -404 | 755 |
| A/A-T/T | 896 | -463 | 2115 | 310 | -234 | 870 |

BCa CI: Bias-corrected and accelerated bootstrap intervals.
