# Supplementary material for: The fat mass and obesity-associated (FTO) gene allele rs9939609 and glucose tolerance, hepatic and total insulin sensitivity, in adults with obesity
Source: PLoS One. 2021 Mar 8;16(3):e0248247. doi: 10.1371/journal.pone.0248247 (PMC7939351; doi:10.1371/journal.pone.0248247)
Supplement: S10 Table — LMM: Linear mixed effects model; CI confidence interval. Intraclass correlation estimates were 0.46 (males) and 0.19 (females). * Significant difference between genotypes (99% bootstrap percentile CI does not include 0). (DOCX) [file pone.0248247.s010.docx]

**S10 Table.** **Parameter estimates and contrasts of time and genotype for each sex for the LMM glucose Rd analyses (**$\boldsymbol{\mu}$**mol/kg_FFM_/min), with 99% bootstrap percentile CI.**

|  |  | **Male** (*n*=30) | | | **Female** (*n*=67) | | |
| --- | --- | --- | --- | --- | --- | --- | --- |
| **Genotype** | Time | Estimate | CI Lower | CI Higher | Estimate | CI Lower | CI Higher |
| T/T | % change clamped-basal | 0.26* | 0.07 | 0.49 | 0.23* | 0.07 | 0.41 |
| A/T | % change clamped-basal | 0.09 | -0.03 | 0.23 | 0.29* | 0.12 | 0.48 |
| A/A | % change clamped-basal | 0.03 | -0.09 | 0.17 | 0.20* | 0.04 | 0.40 |
| A/T-T/T | basal | 1.13 | -1.09 | 3.34 | 1.07 | -1.39 | 3.48 |
| A/A-A/T | basal | -0.81 | -2.64 | 1.00 | -0.61 | -3.18 | 1.94 |
| A/A-T/T | basal | 0.32 | -1.88 | 2.55 | 0.46 | -2.00 | 2.89 |
| A/T-T/T | clamped | -1.16 | -3.42 | 1.07 | 2.32 | -0.19 | 4.77 |
| A/A-A/T | clamped | -1.77 | -3.57 | 0.06 | -2.14 | -4.66 | 0.44 |
| A/A-T/T | clamped | -2.93 | -5.14 | -0.70 | 0.18 | -2.23 | 2.66 |
| A/T-T/T | clamped-basal | -2.29 | -5.45 | 0.85 | 1.25 | -2.23 | 4.73 |
| A/A-A/T | clamped-basal | -0.96 | -3.53 | 1.66 | -1.52 | -5.12 | 2.09 |
| A/A-T/T | clamped-basal | -3.25* | -6.44 | -0.11 | -0.27 | -3.73 | 3.19 |

LMM: Linear mixed effects model; CI confidence interval. Intraclass correlation estimates were 0.46 (males) and 0.19 (females).

* Significant difference between genotypes (99% bootstrap percentile CI does not include 0).
