# Supplementary material for: The fat mass and obesity-associated (FTO) gene allele rs9939609 and glucose tolerance, hepatic and total insulin sensitivity, in adults with obesity
Source: PLoS One. 2021 Mar 8;16(3):e0248247. doi: 10.1371/journal.pone.0248247 (PMC7939351; doi:10.1371/journal.pone.0248247)
Supplement: S11 Table — LMM: Linear mixed effects model; CI confidence interval. Intraclass correlation estimates were 0.34 (males) and 0.18 (females). * Significant difference between genotypes (99% bootstrap percentile CI does not include 0). (DOCX) [file pone.0248247.s011.docx]

**S11 Table.** **Parameter estimates and contrasts of time and genotype for each sex for the LMM glucose MCR analyses (**$\mathbf{m}$**l/kg_FFM_/min), with 99% bootstrap percentile CI.**

|  |  | **Male** (*n*=30) | | | **Female** (*n*=67) | | |
| --- | --- | --- | --- | --- | --- | --- | --- |
| **Genotype** | Time | Estimate | CI Lower | CI Higher | Estimate | CI Lower | CI Higher |
| T/T | % change clamped-basal | 0.27 | 0.08 | 0.51 | 0.26 | 0.07 | 0.49 |
| A/T | % change clamped-basal | 0.11 | -0.02 | 0.26 | 0.33 | 0.13 | 0.57 |
| A/A | % change clamped-basal | 0.02 | -0.10 | 0.17 | 0.21 | 0.01 | 0.44 |
| A/T-T/T | basal | 0.07 | -0.38 | 0.52 | 0.17 | -0.40 | 0.74 |
| A/A-A/T | basal | -0.11 | -0.49 | 0.26 | -0.11 | -0.71 | 0.48 |
| A/A-T/T | basal | -0.04 | -0.49 | 0.41 | 0.06 | -0.52 | 0.63 |
| A/T-T/T | clamped | -0.36 | -0.82 | 0.09 | 0.45 | -0.14 | 1.02 |
| A/A-A/T | clamped | -0.37* | -0.74 | 0.00 | -0.54 | -1.13 | 0.06 |
| A/A-T/T | clamped | -0.74* | -1.19 | -0.28 | -0.10 | -0.66 | 0.48 |
| A/T-T/T | clamped-basal | -0.43 | -1.07 | 0.21 | 0.27 | -0.54 | 1.09 |
| A/A-A/T | clamped-basal | -0.26 | -0.78 | 0.27 | -0.43 | -1.27 | 0.42 |
| A/A-T/T | clamped-basal | -0.69* | -1.34 | -0.05 | -0.16 | -0.97 | 0.66 |

LMM: Linear mixed effects model; CI confidence interval. Intraclass correlation estimates were 0.34 (males) and 0.18 (females).

* Significant difference between genotypes (99% bootstrap percentile CI does not include 0).
