# Supplementary material for: The fat mass and obesity-associated (FTO) gene allele rs9939609 and glucose tolerance, hepatic and total insulin sensitivity, in adults with obesity
Source: PLoS One. 2021 Mar 8;16(3):e0248247. doi: 10.1371/journal.pone.0248247 (PMC7939351; doi:10.1371/journal.pone.0248247)
Supplement: S12 Table — BCa CI: Bias-corrected and accelerated bootstrap intervals. (DOCX) [file pone.0248247.s012.docx]

**S12 Table.** **Parameter estimates and contrasts for combinations of genotype for each sex for glucose infusion rate (GIR) (µmol/kg_FFM_/min), with 99% bootstrap BCa CI.**

|  | **Male** (*n*=30) | | | **Female** (*n*=67) | | |
| --- | --- | --- | --- | --- | --- | --- |
| **Genotype** | Estimate | CI Lower | CI Higher | Estimate | CI Lower | CI Higher |
| T/T | 11.06 | 7.1 | 14.1 | 12.12 | 9.8 | 16.8 |
| A/T | 9.76 | 7.2 | 11.9 | 14.58 | 12.1 | 19.5 |
| A/A | 8.16 | 5.6 | 10.2 | 13.97 | 11.5 | 18.6 |
| A/T-T/T | -1.30 | -5.1 | 3.1 | 2.46 | -1.8 | 7.0 |
| A/A-A/T | -1.60 | -4.7 | 1.8 | -0.61 | -5.1 | 3.9 |
| A/A-T/T | -2.90 | -6.7 | 1.5 | 1.85 | -2.3 | 6.4 |

BCa CI: Bias-corrected and accelerated bootstrap intervals.
