# Supplementary material for: The fat mass and obesity-associated (FTO) gene allele rs9939609 and glucose tolerance, hepatic and total insulin sensitivity, in adults with obesity
Source: PLoS One. 2021 Mar 8;16(3):e0248247. doi: 10.1371/journal.pone.0248247 (PMC7939351; doi:10.1371/journal.pone.0248247)
Supplement: S13 Table — Data presented as median (25th, 75th percentile). Differences between genotype groups determined using Kruskal-Wallis test, * p <0.05, ** p<0.01. P-values are reported with adjustment for ties. aBMI: Body mass index; bFrom DXA; cFat-free mass: Lean mass excluding right and left arms; dTotal fat: Fat mass excluding right and left arms; eAndroid:gynoid fat ratio: Android fat mass/gynoid fat mass; fMeasured with measuring tape. (DOCX) [file pone.0248247.s013.docx]

**S13 Table.** **Anthropometric and body composition measures by genotype group for men younger than 45 years (*n*=19).**

| **Variable** | **T/T** (*n*=6**)** | **A/T** (*n*=7) | **A/A** (*n*=6) |
| --- | --- | --- | --- |
| Age (years) | 28.0 (25, 31) | 38 (28, 41) | 37 (32, 42) |
| BMI^a^ (kg·m_-2_) | 46.0 (44.4, 47.2) | 44.0 (36, 48.6) | 45.6 (41.3, 49.6) |
| Fat-free mass^b, c^ (kg) | 86.4 (82.2, 93.4) | 80.6 (78.3, 90.9) | 87.9 (80.1, 92.0) |
| Total fat^b, d^(kg) | 55.0 (52.0, 58.8) | 44.4 (27.0, 53.5) | 45.6 (38.9, 51.3) |
| Android:gynoid fat ratio^b, e^ * | 0.74 (0.69, 0.78) | 0.85 (0.75, 1.09) | 1.11 (0.98, 1.16) |
| Visceral fat^b^ (g)** | 563 (542, 628) | 914 (414, 1035) | 1295 (1067, 1536) |
| Waist circumference^b^ (cm) | 152.0 (145.0, 153.5) | 145.4 (124.9, 152.2) | 147.3 (137.3, 151.1) |
| Hip circumference^f^ (cm) | 135.8 (134.0, 142.0) | 120.5 (117, 132) | 131.0 (119.5, 134.5) |

Data presented as median (25th, 75th percentile).

Differences between genotype groups determined using Kruskal-Wallis test, * *p* <0.05, ** p<0.01. *P*-values are reported with adjustment for ties.

^a^BMI: Body mass index; ^b^From DXA; ^c^Fat-free mass: Lean mass excluding right and left arms; ^d^Total fat: Fat mass excluding right and left arms; ^e^Android:gynoid fat ratio: Android fat mass/gynoid fat mass; ^f^Measured with measuring tape.
