# Supplementary material for: The fat mass and obesity-associated (FTO) gene allele rs9939609 and glucose tolerance, hepatic and total insulin sensitivity, in adults with obesity
Source: PLoS One. 2021 Mar 8;16(3):e0248247. doi: 10.1371/journal.pone.0248247 (PMC7939351; doi:10.1371/journal.pone.0248247)
Supplement: S14 Table — Values are median (25th, 75th percentile). aP-values derived from two-sample Wilcoxon rank-sum (Mann-Whitney) test for differences between males and females. bMatsuda index: Indexofwholebodyinsulinsensitivity=10000(fastingglucoseconc⋅fastinginsulinconc)⋅(meanglucoseconc⋅meaninsulinconc) cFFM: Fat-free mass is lean mass excluding right and left arms measured by DXA. dHIR: Hepatic insulin resistance index = basal glucose Ra · basal insulin. (DOCX) [file pone.0248247.s014.docx]

**S14 Table.** **Median for glucose tolerance and insulin sensitivity variables for males and females independent of genotype.**

| **Variable** | **Males** | **Females** | ***p*-value^a^** |
| --- | --- | --- | --- |
| **Meal test** | ***n*=30** | ***n*=67** |  |
| Glucose 0min (mmol·L^-1^) | 5.60 (5.17, 5.86) | 5.45 (5.12, 5.77) | 0.1823 |
| Glucose 30min (mmol·L^-1^) | 7.48 (6.92, 7.90) | 7.07 (6.51, 7.68) | 0.0656 |
| Glucose 150min (mmol·L^-1^) | 6.01 (5.33, 6.51) | 5.55 (5.16, 6.10) | 0.0478 |
| Insulin 0min (pmol·L^-1^) | 163.71 (124.88, 215,72) | 116.40 (87.19, 147.79) | <0.001 |
| Insulin 30min (pmol·L^-1^) | 643.34 (551.81, 1048.80) | 606.88 (409.13, 812.44) | 0.1073 |
| Insulin 150min (pmol·L^-1^) | 394.48 (267.76, 545,77) | 303.07 (223.75, 407.78) | 0.0323 |
| Matsuda insulin sensitivity index^b^ | 1.74 (1.46, 2.50) | 2.69 (1.99, 3.76) | <0.001 |
| **Clamp** | ***n=*25** | ***n=*54** |  |
| EGP basal (µmol·kg_FFM_^-1^·min^-1^)^c^ | 14.39 (13.81, 15.15) | 15.72 (14.64, 16.43) | <0.001 |
| EGP clamped (µmol·kg_FFM_·min^-1^)^c^ | 5.84 (4.56, 6.23) | 4.33 (3.55, 6.28) | 0.0313 |
| EGP % decrease | 62.26 (55.81, 69.90) | 72.29 (58.81, 77.45) | 0.0037 |
| Glucose Rd basal $(\mu$mol·kg_FFM_^-1^·min^-1^)^c^ | 14.51 (13.92, 15,29) | 15.85 (14.80, 16.60) | <0.001 |
| Glucose Rd clamped $(\mu$mol·kg_FFM_·min^-1^)^c^ | 16.29 (14.42, 17.77) | 18.97 (17.22, 20.50) | <0.001 |
| Glucose Rd % increase | 6.84 (-0.13, 22.54,) | 18.21 (10.09, 29.39) | 0.0120 |
| Glucose MCR basal (ml·kg_FFM_·min^-1^)^c^ | 2.73 (2.64, 2.96) | 3.15 (2.93, 3.36) | <0.001 |
| Glucose MCR clamped (ml·kg_FFM_·min^-1^)^c^ | 3.03 (2.68, 3.49) | 3.81 (3.42, 4.34) | <0.001 |
| Glucose MCR % increase | 7.65 (-0.67, 23.03) | 20.29 (-9.55, 31.52) | 0.0065 |
| GIR (µmol·kg_FFM_·min^-1^)^c^ | 9.84 (7.50, 10.97) | 13.19 (9.98, 16.34) | <0.001 |
| HIR^d^ | 1681.6 (1396.96, 2833.53) | 1764.91 (1164.77, 2037.14) | 0.2003 |
| Glucagon 100 min (ng·L^-1^) | 88.71 (72.90, 113.96) | 73.75 (59.84, 83,16) | 0.0011 |
| Glucagon 240 min (ng·L^-1^) | 86.59 (71.95, 104.93) | 65.32 (56.85, 77.88) | <0.001 |
| $\Delta$ Glucagon (ng·L^-1^) | -8.25 (-17.24, 4.03) | -8.18 (-17.13, 4.03) | 0.7624 |

Values are median (25th, 75th percentile).

^a^*P*-values derived from two-sample Wilcoxon rank-sum (Mann-Whitney) test for differences between males and females.

^b^Matsuda index: Index of whole body insulin sensitivity

$$=\frac{10000}{\sqrt{(\text{fasting glucose conc}\cdot\text{fasting insulin conc})\cdot(\text{mean glucose conc}\cdot\text{mean insulin conc})}}$$

^c^FFM: Fat free mass is lean mass excluding right and left arms measured by DXA.

^d^HIR: Hepatic insulin resistance index = basal glucose Ra · basal insulin.
